# Shifting echo chambers in US climate policy networks

**DOI:** 10.1371/journal.pone.0203463

**Published:** 2018-09-14

**Authors:** Lorien Jasny, Amanda M. Dewey, Anya Galli Robertson, William Yagatich, Ann H. Dubin, Joseph McCartney Waggle, Dana R. Fisher

**Affiliations:** 1 Department of Politics, University of Exeter, Exeter, United Kingdom; 2 Department of Sociology, University of Maryland, College Park, MD, United States of America; 3 Department of Sociology at the University of Dayton, Dayton, OH, United States of America; 4 Center for Climate Change Communication, George Mason University, Fairfax, VA, United States of America; 5 Independent Researcher, New York, NY, United States of America; 6 Independent Researcher, Washington, DC, United States of America; University of Waikato, NEW ZEALAND

## Abstract

Although substantial attention has focused on efforts by the new Administration to block environmental policies, climate politics have been contentious in the US since well before the election of Donald Trump. In this paper, we extend previous work on empirical examinations of echo chambers in US climate politics using new data collected on the federal climate policy network in summer 2016. We test for the similarity and differences at two points in time in homophily and echo chambers using Exponential Random Graph Models (ERGM) to compare new findings from 2016 to previous work on data from 2010. We show that echo chambers continue to play a significant role in the network of information exchange among policy elites working on the issue of climate change. In contrast to previous findings where echo chambers centered on a binding international commitment to emission reductions, we find that the pre-existing echo chambers have almost completely disappeared and new structures have formed around one of the main components of the Obama Administration’s national climate policy: the Clean Power Plan. These results provide empirical evidence that science communication and policymaking at the elite level shift in relation to the policy instruments under consideration.

## Introduction

With the election of Donald Trump as the 45^th^ President of the United States, American environmental politics have become even more contentious [1]. The issue of climate change has been a central focus of debate, with the new Administration halting efforts to monitor and regulate greenhouse gases. Despite a well-documented scientific consensus on the causes and drivers of global climate change, the President and a number of his appointees are well known for questioning the science of the issue. In fact, the President’s first appointee to run the Environmental Protection Agency led the state of Oklahoma’s case against the Obama Administration for trying to implement the Clean Power Plan to reduce greenhouse gas emissions nationwide. While scientists continue to warn decisionmakers about the need to act [2–8], the political debate remains polarized [9–11]. In August 2015, President Barack Obama finalized his Clean Power Plan Executive Order, which would regulate emissions from power plants. Within days, governors and attorneys general from a number of states announced their intention to oppose the Plan on the grounds that it was federal overreach into state affairs [12].

Recent research illustrates that the structure of policy actor networks can explain how information flows in the political sphere and demonstrates how these structures can lead to real-world policy outcomes [10,13,14]. Specific to climate policy, scientists have looked to the news media and communications [15–17], the rise of the conservative counter-movement [9,18], and the polarization of the issue within the US Congress [10,19,20] to explain how an issue of such wide scientific consensus can lead to sustained political gridlock. A handful of studies have specifically studied climate policy networks ([21–24] see also [25]). In their study of Swiss climate policy, Ingold and Fischer look longitudinally across three time periods, finding that common beliefs among actors and formal power structures explain much of the collaboration networks among policy actors [22].

**Fig 1:**
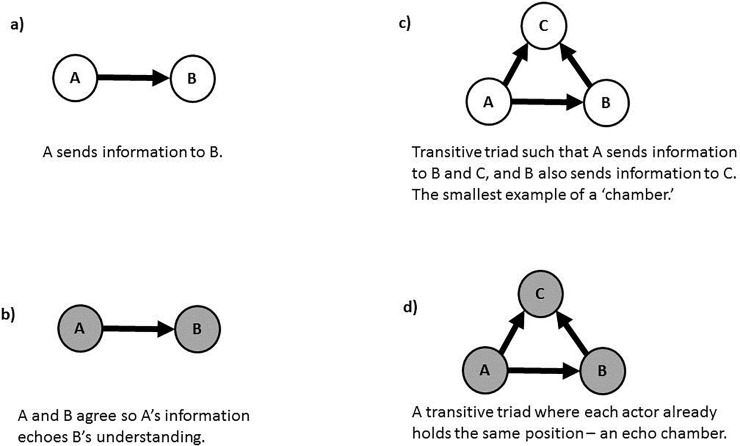
Structural and attitudinal components of an echo chamber.

In this paper, we look at two periods in time to understand climate policy in the United States. Instead of focusing on collaboration, however, we build on previous work on echo chambers to understand information diffusion among the climate policy network [21]. In this previous study, echo chambers were operationalized as the combination of two components: the sharing of information between two actors who have the same outlook or opinion on a relevant issue, or the "echo;" and the information from the same source reaching the same endpoint via multiple direct and indirect paths, or the "chamber" [21]. The “echo” is the sending of information from a source to a recipient who holds the same stance on a particular piece of policy. This stance or view is represented by the shading of the circles (representing the actors) in Fig 1B (as opposed to the clear circles in 1a). The “chamber” mechanism, in contrast, has information from the same source reaching the same recipient via multiple different paths. The smallest structural configuration that would depict this process is the transitive triad. In this structure, information passes from actor *A* to actor *C* through a direct tie, but also indirectly through actor *B* (such that *B* receives information from *A*, and *C* receives information from *B*; see Fig 1C).

This structure combines the transitive triad, which Carpenter has posited promotes trust in information networks [13], with the clustering of like-minded actors, structures that were shown by Klar and Shmargad in an experiment to promote less learning and exposure to diverse viewpoints than random networks [26]. Williams et al. also found transitive triads to promote polarization in an observational study [27].

In previous work, this operationalization of echo chambers was applied to data collected from political elites working on climate policymaking in the US in 2010. Here, again using Exponential Random Graph (ERG) models, we test empirically whether or not echo chambers have shifted to form around a more recent climate policy. This analysis enables us to answer three related research questions: Are there echo chambers in the US climate policy network in 2016 structured around the Clean Power Plan? How stable are the policy positions in our network of elites engaged in climate politics? And, how have structural properties of the federal climate policy network shifted from 2010 to 2016?

## Materials and methods

To show that an echo chamber exists, we must first demonstrate that these transitive triads contain policy actors with the same viewpoint, and second that they play an important role in the network. We examine the information networks that supplied members of the climate policy community in the United States with research, advice, and perspectives on the issue of climate change in 2016. Our network comprises the set of policy actors in our sample who responded to our 2016 survey (49 in total) and all reported directed communication within this population.

### Constructing a data set and sampling

Consistent with previous research studying echo chambers in climate policy networks, we began by creating a comprehensive dataset of elite United States climate policy actors engaged in policymaking during the period of our study [21]. A dataset was assembled from three publicly available sources. We began with a list of all the policy actors who participated in climate-related hearings in the United States Congress during the two sessions prior to when data collection began: the 112^th^ session (January 2011-January 2013) and the 113^th^ session (January 2013- January 2015). Although it would have been ideal to include a list of the speakers who were participating in the on-going 114^th^ session of the US Congress as well, since data collection was taking place in the middle of this particular session, a complete list of hearings and participants was not available.

Next, using the House [28] and Senate [29] Lobbyist Disclosure Act databases, we added a field to note all lobbyists who were registered to lobby on climate issues during each respective period. Finally, we added a field to indicate who on the list had participated in the international climate change negotiations (COP-21) in Paris in December 2015 [30]. Participants were neither added nor removed from the dataset based on their participation in the international negotiations or registration as climate lobbyists. Rather, participation in these two additional arenas were tallied together so each actor was scored based on their level of engagement within the climate policymaking arena. By drawing from these varied sources that span the two previous sessions of the Congress, we were able to assemble a dataset that measured sustained engagement in the climate policy network over the years leading up to our period of data collection.

Next, actors in this dataset were ranked according to the degree to which they participated in hearings, international negotiations, and on lobbyist registries (if they were non-state actors). Testimonies were weighted such that multiple appearances before Congress indicated greater participation. Our sample includes all actors who participated more than once in this "climate policy arena." In some cases, policy actors participated in climate-related Congressional Hearings more than once, but in other cases, the actors participated in the Congress only once but also participated in the climate negotiations and/or were registered to lobby on the issue.

In contrast to the sampling period for the piece by Jasny and colleagues when climate-related legislation was working its way through the US Congress [21], this four-year period saw much less climate-related legislative action. As a result, only 83 policy actors met the sampling criteria and were identified as central climate policy actors. Given this lower number, we then added into the sample four additional policy actors who had ranked high in our 2010 sample and had also been active on the issue during the 114^th^ session of the US Congress (which was ongoing during our data collection period): Senator James Inhofe of Oklahoma; the American Council for an Energy-Efficient Economy; World Resources Institute; and the Pew Center on Global Climate Change, which was renamed the Center for Climate and Energy Solutions in 2011. We also added the Intergovernmental Panel on Climate Change, which was included in our 2010 sample and is the “international body for assessing the science related to climate change” [31]. In total, our sample of the 2016 climate policy network included 88 policy actors who were especially influential in the federal climate arena following the ‘events based’ approach to determining network boundaries [32]. In network analysis these kinds of sampling issues are called ‘boundary’ problems and are routinely encountered [33].

Like previous studies [21,34], we do not generalize our findings to the entire population of actors. Rather, our goal is to understand the dynamics of the most central actors in the climate policy network in the United States at the federal level with the understanding that the processes at work in this sample are unlikely to resemble the dynamics outside this sample [35]. We maintain that networks among these central actors are critical for understanding US federal climate policy.

It is important to note that our data set, like those of others working from a policy network perspective [25,36–38], includes actors with all types of organizational affiliations, including both state and non-state actors. We operationalize non-state actors as those who are not employed by the government; non-state actors include business and trade union representatives, members of the environmental and climate policy teams at NGOs, and university scientists. State actors include both climate-focused staff people from Congressional offices, and employees working in the Administrative branches of federal agencies. The authors coded all respondents into one of these categories based on where they worked during the sampling period. Not only does this sampling method provide a range of actor types, but it notably also provided us with actors who represent a diversity of ideological positions on the issue of climate change in the United States.

### Data collected

The data analyzed in this paper were collected in Summer 2016. We compare these data to data that were collected in 2010 [21]. Data collection took place in 2010 during a period when climate policy was working its way through the US Congress: The American Clean Energy and Security Act, sponsored by Representatives Henry Waxman and Edward Markey, had been passed by the House of Representatives in summer 2009; during summer 2010, while the research team was in the field surveying respondents, there were efforts to pass a Senate companion bill. The American Power Act was released by Senators Kerry and Lieberman with input from Senator Graham in May 2010 (for details, see [39]). In July 2010, however, Senate Majority leader Reid dropped regulating greenhouse gas emissions from the energy bill due to lack of bipartisan support (for details, see [40]).

In contrast to this highly contentious period in the Congress in 2010, the 2016 data collection period occurred during a waiting period for President Obama's Clean Power Plan. The Clean Power Plan was an executive order designed to regulate emissions from power plants in the US through the Environmental Protection Agency. Implementation of the Plan had been stayed by the US Supreme Court in February 2016 until the legal challenges to the program had been concluded [41]. A final decision from the courts regarding the future of the Clean Power Plan was expected after the 2016 election.

As we were asking political elites’ about their organization’s official/public views, oral consent was attained as approved by the UMD IRB (Protocol #878998). For the 2010 Survey, oral consent was approved by the Columbia University IRB (# IRBAAAG2612) and University of Maryland (IRB Protocol #10–0751). See [21] for additional details of the 2010 survey; the specifics of the 2016 survey follow. The 88 policy actors identified in our sample were contacted to participate in our study as they represent the core of political elites that have the most influence over the policy process. Data were collected through in-person meetings in the Washington, DC metropolitan area whenever possible. Contact was initially made via email and telephone with all of the policy actors in the sample that had been included in previous rounds of research [21,42]. For offices that had never been contacted before, the researchers aimed to contact a representative who had participated in Congressional Hearings during the sampling period. In some cases, participants in the study were asked if they could provide a contact for offices with whom they collaborate. Respondents were offered no incentives to participate in the study.

In these in-person settings, actors were interviewed and administered a written survey. After the surveys were filled out by respondents, the research team manually entered the data into Qualtrics. For policy actors who were not available to meet or who were located outside of the DC area, interviews were conducted over the phone and surveys were conducted through Qualtrics online. The survey data are the focus of this paper. In total, survey data were collected from 50 policy actors in our sample, representing a 57% response rate. Although this response rate for the 2016 wave is somewhat lower than the 64% response rate for the 2010 data [21], it is consistent with other studies of communities of political elites [43,44]. Two of the respondents were from different branches of the EPA but these were collapsed so that the agency was not doubly represented in the network leaving 49 total actors in our analyses. Of the two EPA respondents, we selected the responses from the more senior interviewee, however the results and conclusions presented here do not change if the more junior interviewee is selected. Although a relatively small sample, these data represent a unique dataset from the most central policy actors working on climate change in the US during this period of time. Moreover, the size of our sample is not unusual for the policy networks literature (For examples of networks of similar sizes, see [25,43,44]).

Even though respondents included actors from across the political spectrum and from all types of organizational affiliations, we observed some obvious differences between the respondents and the non-respondents in our study. Specifically, the offices of Representatives and Senators in the US Congress again had a much lower response rate than the other types of political actors in 2016 (20% versus a response rate of around 57% for the sample overall). This response rate is consistent with that for elected officials in the US Congress in the previous work by Jasny et al. [21].

### Survey instrument

**Fig 2:**
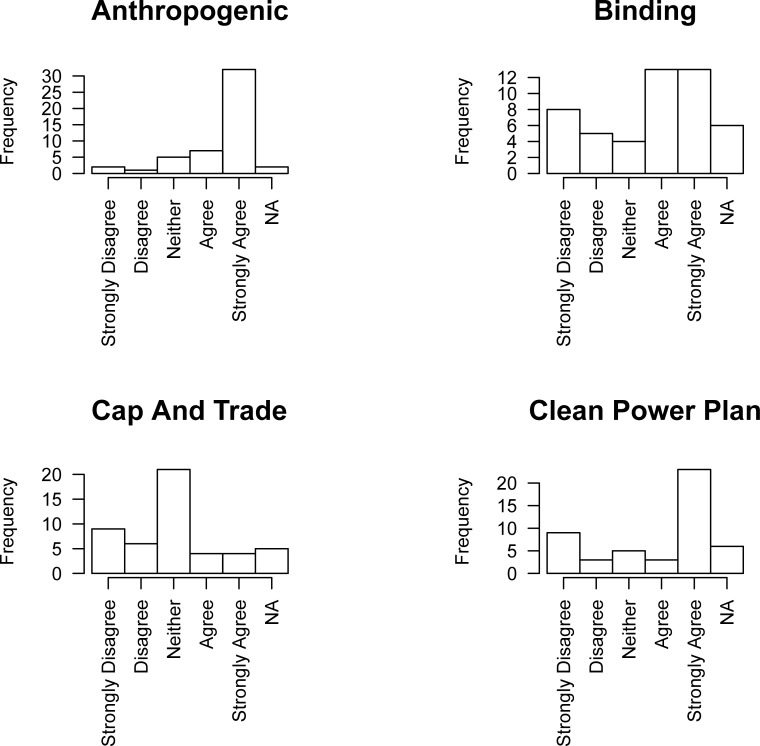
Frequencies for 2016 attitudinal data.

The survey itself was comprised of three types of questions. Attitudinal questions asked participants to indicate on a scale of 1 to 5—where 1 indicated strong disagreement, 5 indicated strong agreement, and 3 indicated neutrality—their positions on statements that were deemed politically salient during the data collection period. This paper presents analysis of three attitudinal questions that were the focus of previous analysis [21]: *Human activities are an important driver of current global climate change* (2016 survey Question 1, item 3: referred to in the paper as Anthropogenic), *Emissions trading (cap and trade) is the best option for reducing US GHG emissions* (2016 survey Question 2, item 2: referred to as Cap and Trade), and *There should be an international binding commitment on all nations to reduce GHG emissions* (2016 survey Question 2, item 6: referred to as Binding). We also include analysis of the question, *The Clean Power Plan should be implemented by every state* (2016 survey Question 2, item 7: referred to as CPP), as the Clean Power Plan had been stayed by the Supreme Court but at the time of data collection, it was expected to be implemented eventually. Distributions of responses for these four items are presented in Fig 2.

Most important for the purposes of the present research, this survey asked three network questions. Each of the 88 policy actors in our sample was listed in alphabetical order by actor type, and each respondent was presented with three iterations of this list. Participants were then asked to respond to three network questions. Specifically, they were asked to indicate, in order: those actors or organizations whom they identified as their sources of expert scientific information about climate change, those actors or organizations they collaborate with on a regular basis, and those actors or organizations whom they perceived to be most influential in climate politics, in any ideological direction. The first network question—reporting sources of expert scientific information—makes up the foundation of this research. A copy of the survey, as well as the public dataset and a codebook, can be found at: http://drfisher.umd.edu/CCP.html.

The authors also collected data on organizational and structural characteristics (such as the age of the organization, the organization’s number of employees, and so forth) from a systematic review of the websites and published materials of each policy actor. Due to their unique nature among other types of actor, many of these variables are inappropriate for the Congressional offices in our sample, and are therefore missing.

### Exponential Random Graph Models

We test for echo chambers and a variety of other configurations using Exponential Random Graph Models (ERG). ERG models use simulation methods to address the bias introduced in regression models by the interdependence of network ties, and are increasingly being used in the study of policy networks [45,46,25,47]. Evidence of echo chambers (Fig 1D) would indicate directed information flow among this network of policy actors working on climate change that is based on the combination of agreement on a particular policy position (Fig 1B), and the structure of a transitive triad (Fig 1C). Our analytic technique enables us to test if information diffusion takes place randomly or if perspectives on a specific policy instrument (such as the Clean Power Plan) determine who gets information from whom. Finding evidence of echo chambers in American climate politics provides evidence that information is being cherry picked by policy actors, as well as the degree to which the information is being amplified through direct and indirect communication channels.

**Fig 3:**
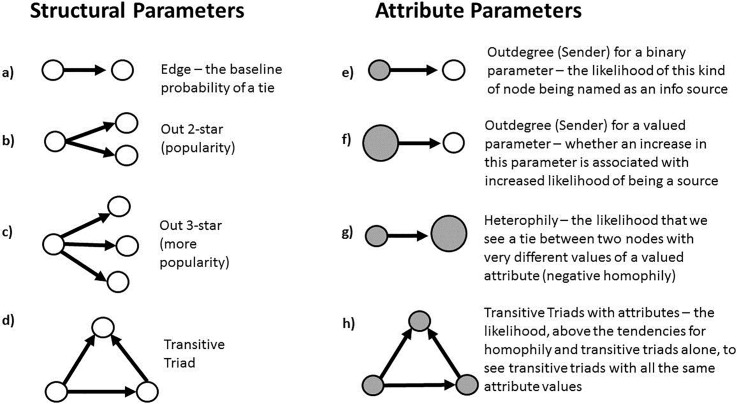
Terms used in the ERG models.

The terms included in the models are presented in Fig 3 and are the same as those used in Jasny et al. [21]. The results of an ERG model are presented in log-odds form. The edges term (Fig 3A) acts like an intercept–it is interpreted as the base rate of tie formation given every other term in the model. Terms for out-star popularity (Fig 3B and 3C) look at whether certain organizations were popularly named as sources of scientific information (the direction of the arrow follows the information from the source to the respondent). As in previous work [21] we operationalize the ‘chamber’ of an echo chamber to be a transitive triad (Fig 3D). Transitive triads provide evidence of simultaneous direct and indirect information diffusion. We also include attribute terms: Fig 3E and 3F show the terms for certain types of organizations being more or less popular as sources of information depending on their attributes. The attributes for our model include the organization type (categorical), lobbying budget and FTE which capture organizational resources, and the attitudinal responses to the questions of interest in the survey (see Fig 2). Homophily is represented in Fig 3G and is the tendency for organizations to go to those for information who share their belief. This is the ‘echo.’ Beliefs are measured based on policy actors’ response to questions about their policy position. The combination of the ‘echo’ and ‘chamber’ in Fig 3H is the full operationalization of the echo chamber. ERG models permit the testing of all of these terms simultaneously in the model predicting edge formation [48]. The ERG models were fit using the Statnet (v2.3.2) software package for the R (v3.2.2) programming language [49]. We constrained the simulations on the indegree distribution to match the method used by Jasny and colleagues [21].

### Missing data

We made a number of decisions about handling missing data. First, non-respondents were removed from the network, reducing our size from the 88 actors in the roster to the 50 respondents, which resulted in 49 actors (to restate, the two EPA respondents were collapsed). While this approach is clearly not an ideal method, it is standard in network analysis [45,50] and appropriate missing data methods are still being developed [35]. Additionally, we had cases where respondents left questions blank. In order to use the exponential random graph model (ERGM) attribute methods in Statnet, we could not leave any continuous entries missing (continuous covariate terms are a combination of the value for sender and receiver, as this omission would have resulted in far too much missing data to calculate an accurate value for the covariate effect). The 2010 data had more missing data than 2016 with 4 and 2 missing entries for Anthro respectively, 12 and 6 for Binding, 9 and 5 for Cap and Trade, 11 and 4 for the number of full time employees, 15 and 3 for budget, and finally 6 for CPP which was only asked in 2016. We handled these missing data in two ways: first, by replacing them with the average value, and second by imputing values using predictive mean matching using the Hmisc package for R (v4.1–1, [51]). Ten separate sets of values were imputed and ERGMs run on each set. The results were then combined following Raghunathan ([52] see Chapter 4). However, for each of the echo chamber terms we treated NAs as a separate category and removed them from the counts so that echo chambers among the NAs were not incorporated in the statistics. The average and imputed results are shown separately in our model results but are consistent in significance and interpretation.

## Results

**Fig 4:**
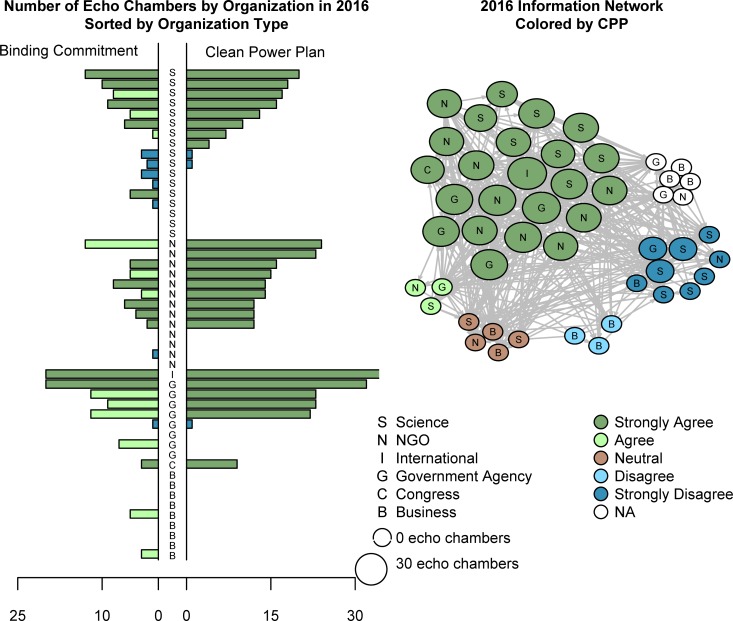
Echo chambers in 2016: A histogram by organization type for the numbers of echo chambers where all hold same view on 'Binding Commitment' (left), A histogram by organization type for the numbers of echo chambers where all hold same view on 'Clean Power Plan' (middle), the 2016 network showing view on 'Clean Power Plan'.

Fig 4 presents the descriptive results of three components of our data. First, the left-hand side of the bar chart displays the number of echo chambers for policy actors in our analysis based on their sources of “expert scientific information” and their responses to an attitudinal question that asks them to identify their organization’s position from “strongly agree” to “strongly disagree” on the statement: *There should be an international binding commitment on all nations to reduce GHG emissions*. This variable indicates the policy actor’s position on an international climate treaty and we refer to this variable as Binding. The echo chambers discussed in [21] centered on this policy instrument, although the data presented in Fig 4 is from the 2016 survey. Second, the right hand side of the bar chart shows the number of echo chambers for policy actors in our analysis based on their sources of “expert scientific information” and their responses to an attitudinal question that asks them to identify their organization’s position from “strongly agree” to “strongly disagree” on the statement: *The Clean Power Plan should be implemented in every state* (the policy instrument intended to regulate emissions in 2016). We refer to this variable as CPP. Thus, the horizontal axis measures the number of transitive triads where all members agree on Binding (left) versus the number where they agree on CPP (right). The organizations are ordered horizontally by organization type (see key to the right) and then in order of the number of CPP echo chambers. We see clear differences between organization types and numbers of echo chambers, with no CPP echo chambers among Business respondents (‘B’), but many among each of the other types of organizations. There are clearly more echo chambers around the CPP (396) than the Binding (206) policy instrument. Where the echo chambers in 2010 were found at all levels of agreement with Binding, in 2016 they are split between ‘Agree’ and ‘Strongly Agree.’ The echo chambers around CPP are, with three exceptions, exclusively around ‘Strongly Agree’. A comparison of the organizations common to the two samples follow, but in fundamentally thes echo chambers observed in 2016 are different from those observed in the 2010 data [21]. In other words, during the period of our research in 2016, which was after the Obama Administration had already signed on to the Paris Agreement, the Clean Power Plan had much more policy salience (For an overview, see [53]). Finally, the right hand side of the diagram presents a graphical depiction of the echo chambers in the later network colored by their attitudes towards CPP, showing the concentration of ties and therefore echo chambers among that policy position. Thus in 2016, while echo chambers form less frequently around Binding Commitment, they have formed in larger numbers and with greater agreement around the CPP.

A direct comparison of the full 2010 and 2016 data is complicated by the changes in the policy actors identified to participate in the study. The six-year period from 2010 to 2016 marked a shift in institutional strategy on climate change as organizations developed responses to both international and national developments. We see this change represented in the population of actors who met our sampling criteria, changes in their attitudes on policy, as well as changes in the network of information ties. Only 27 policy actors from the 100 in our 2010 sample were included in 2016 and only 19 of those policy actors participated in the study, making longitudinal network modeling impossible unless we restrict to just those organizations [54]. While we compare the samples from 2010 and 2016 to get a sense of the change in the two points in time, the analysis performed in this paper is cross-sectional instead of longitudinal, which allows us to use the full samples from 2010 and 2016 rather than the smaller intersection of the two.

**Fig 5:**
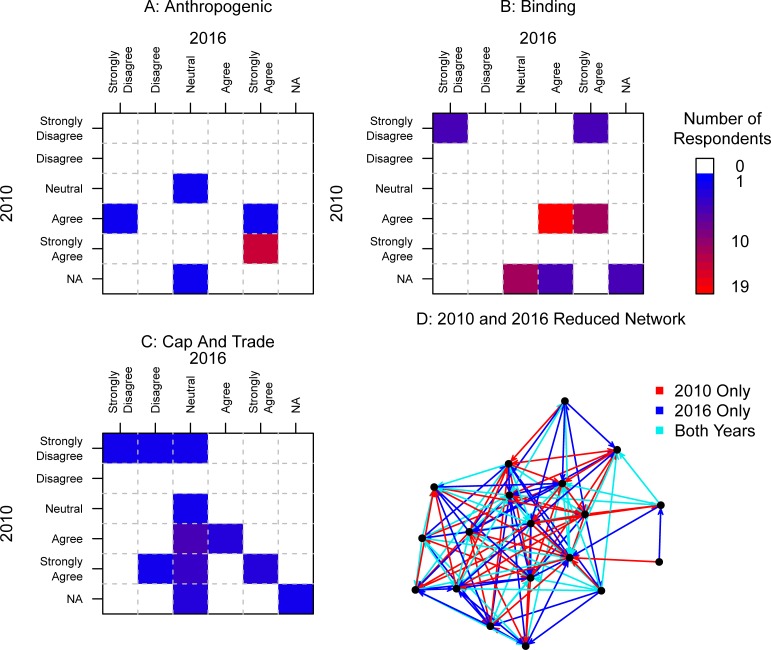
Comparison of 2010 and 2016 data by the information network and the repeated attributes (Anthropogenic, Binding Commitment, Cap and Trade).

Fig 5 presents heat maps comparing the policy positions of the actors who were included in both the 2010 and 2016 analyses and the subnetwork of the 19 actors who responded to both surveys. “Heat” in this context represents the concentration of actors for each response on our attitudinal scale. These 19 actors were remarkably stable with regard to whether they believed that *Human activities are an important driver of current global climate change* (referred to as Anthropogenic); the observations are either on the diagonal in the figure (meaning no change in opinion) or slightly off (for example, one actor switched from “Strongly Agree” to “Agree [Fig 5A]). Compare these results to the results of the other policy positions. The first is Binding, discussed above, and the second is a policy statement we refer to as Cap and Trade: *Emissions trading (cap and trade) is the best option for reducing US GHG emissions*. Both showed more change than that of Anthropogenic, but still not huge changes or shifts in perception. Since the Clear Power Plan had not been introduced in 2010, there are no comparable data for this policy position.

There were 7 total changes in opinion between 2010 and 2016 on Binding (not including NAs) and one actor switching from “Strongly Agree” to “Strongly Disagree” (Fig 5B). Cap and Trade (Fig 5C) had the most change with 10 policy changes, but none were as extreme as the reversal in Binding. Fig 5D displays the reduced sample of 19 actors who responded to both surveys. It is difficult to discern any patterns of change in the figure; for that we will turn, after some basic descriptives, to the statistical modeling. The network of information flow between these organizations also changed greatly with 41 of the 95 ties in the 2010 network no longer mentioned in 2016 (43%) and an additional 63 ties added for an overall change in the network of 30% of the possible ties. The subsample of the 19 repeated surveys shows us that this period was marked by some limited change in policy stance and the severing of many of the information ties found in the previous data with more new ties reported in 2016. However, these small changes had big consequences for the formation of echo chambers: of the 14 Binding echo chambers in this 19 organizational subsample, only one echo chamber stayed constant in structure and agreement from the 2010 to the 2016 data. We therefore see that the combination of policy agreement (the ‘echo’) and structural composition (the ‘chamber’)–can be more sensitive to change than either component individually. To understand the statistical relationship between structural properties and agreement on policy position, we turn to cross-sectional modeling of the two datasets.

**Fig 6:**
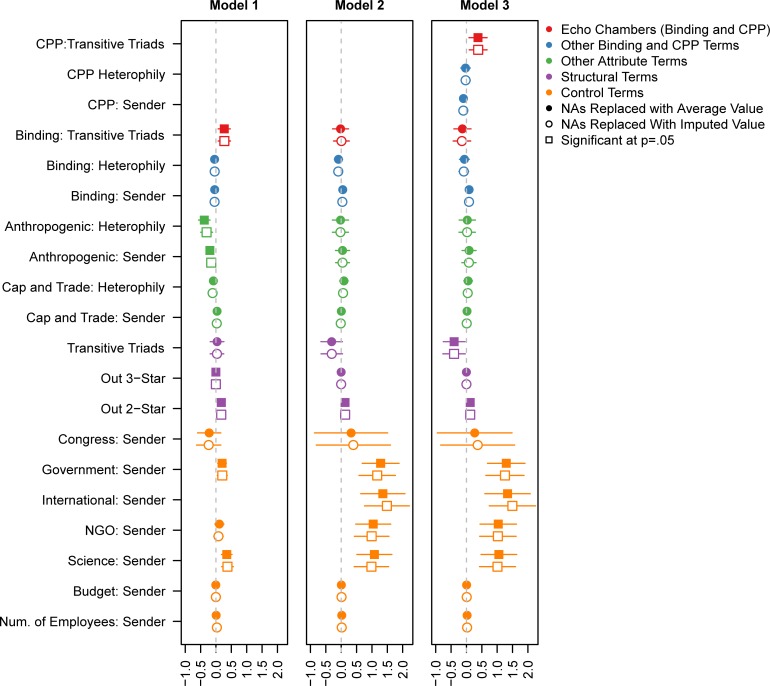
Results from 3 ERG models; Model 1 displays the results for the 2010 data, Model 2 repeats Model 1 but with 2016 data, and Model 3 adds terms for Clean Power Plan (CPP).

Fig 6 presents the results of the ERG simulation methods to test for the presence and significance (relative to tie formation) of echo chambers holding other structural tendencies constant. The coefficients are logged odds ratios. Thus, the logged ratio of any given tie existing in the network can be expressed as a sum of the relevant terms in the model. Model 1 presents the results from the 2015 paper (based on data collected in 2010). Model 2 reprises Model 1 using the more recent data collected in summer 2016. Model 3 adds terms related to the Clean Power Plan.

Results from replacing NAs with average values are presented with solidly colored symbols and results using imputed data with symbols with a white center; there are no significant differences between these two methods of handling the missing data. Two structural terms are statistically significant across all three models: the significant out 2-star term shows that certain organizations are popular sources of information, and the significant sender terms for government agencies and scientific organizations indicate that these organization types are more frequently listed as sources of information compared to the reference group of business organizations. In 2010, only an effect for echo chambers around Binding was found (Model 1). In 2016, however, the significant echo chambers have shifted to form around the Clean Power Plan (Model 3). It is worth noting that, in addition to the significance of these echo chambers in 2016, we find a negative effect for additional “chambers” (or transitive triads) without the echo, indicating the strength of these echo chambers (see Fig 3). Compared to business groups, NGOs and the one international organization–the Intergovernmental Panel on Climate Change (IPCC)—are significantly more often cited as a source of expert scientific information in 2016. Given its status as the international body responsible for assessing the science related to climate change, it is not particularly surprising that the IPCC is slightly more likely to be cited as a source than the actors in every other category.

To interpret the significance of the echo chambers around the CPP, we can think about the likelihood of an actor citing another actor as a source when a) this tie would add one echo chamber to the network and b) when this tie would add a transitive triad but not an echo chamber. If both of these organizations are businesses (the reference category), and in addition this tie adds one out-2 star (a term for preferential attachment or popularity) to the network, then in case (a) the log-odds of this tie being formed is just the sum of the three relevant coefficients (the significant terms for out 2-star, transitive triads, and transitive triads around the CPP) which is 0.102. Converting this number from log-odds yields a probability of 52.5% of this tie occurring. If, however, the tie only added a transitive triad but not an echo chamber, then we leave out the coefficient for CPP transitive triads from the sum. The resulting probability is 43% (from a log-odds of -0.275). Consequently, we observed an increase of 9.5 percentage points. These findings provide clear evidence that strong support for the Clean Power Plan was driving where policy actors went for climate information: more policy actors strongly agreed with the policy and they transmitted information directly and indirectly among themselves, thus amplifying their policy perspective. The combination of finding significance for the full ‘echo chamber’ term, rather than the separate terms for homophily (the ‘echo’) and transitive triads (the ‘chamber’), shows that these two separate mechanisms are significantly combined in our empirical data.

**Fig 7:**
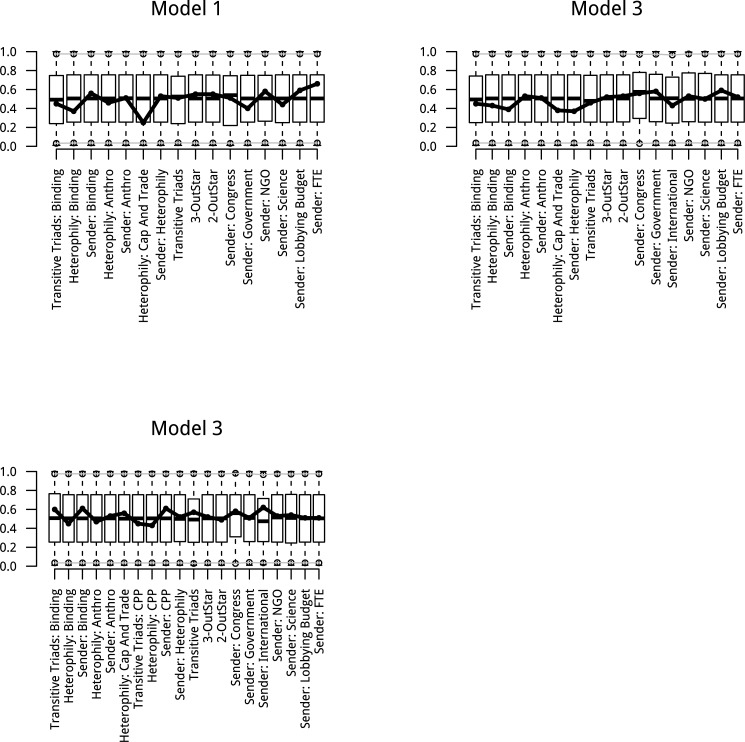
Model adequacy checks for ERG models.

Model convergence and goodness-of-fit is essential for the interpretability of the model. The model adequacy check examines how well the simulation mixes over the sample space and whether these simulated networks produce normal distributions centered at the empirical values for each statistic in the model. Goodness-of-fit measures were run for each model which simulated 10,000 networks from the model to compare to the empirical values. Quantiles for these simulated values are presented for each term in each model in Fig 7. Models are deemed interpretable when the p-values are close to 1 indicating that the simulated models capture the statistics of interest. The smallest p-value from the goodness-of-fit diagnostics was 0.74 indicating good model fit.

## Discussion

As was noted by Jasny and colleagues [21], high levels of transitivity can have a deleterious impact on networks of information transmission. In other words, “the repeated nature of the ties may give members the impression that an issue is decided when there continues to be debate” [21]. Within contentious climate politics, echo chambers may also amplify divergence from the consensus. A modicum of dissenting voices can appear to represent a substantial number of dissenters when amplified through an echo chamber. Likewise, the echo chamber may also amplify convergence. It is important to note that echo chambers themselves are value-free and apolitical; their impacts on policy discussion and debate are an effect of the political context and the ideological positions of the actors within them. In the context of federal US climate politics, the results presented in this paper provide evidence that echo chambers shift to focus on specific policy instruments, in this case the Clean Power Plan. It is also worth highlighting that, in contrast to the results from 2010 that found echo chambers amplifying divergence from consensus, in 2016 echo chambers amplified convergence among members of the climate policy network. Although these results show that there was a very low level of divergence across the policy network at this point, it did not lead to a successful policy outcome for the Clean Power Plan. In other words, these results provide a clear reminder that we should not over-interpret the political significance of these structures; while the Clean Power Plan was prominent in the agenda of the Obama Administration, despite the agreement and the echo chambers observed in our results from this research, the Plan is no longer politically viable under the Trump Administration.

This paper is a first step in analyzing information diffusion in policy networks over time. Although it does not provide time-series analysis, it compares static network data collected during two points in time with limited overlaps. Expansions on this work will engage with temporal data to explore the nascence and formation of echo chambers (asking, for example, which comes first: the echo or the chamber?) drawing on current research regarding influence versus selection mechanisms in homophily [54]. However, the findings in this paper, which compare the 2010 and 2016 cross-sectional data, provide important conclusions about the development and maintenance of networks of expert scientific information used by political elites. We see a sustained focus on echo chambers and that these structures decrease around views no longer at the center of the policy process (the content of an international agreement as measured in Binding Commitment) and form around those that are more policy salient (the Clean Power Plan). In both years, there was significance for these structures above and beyond the tendency for either the echo (homophilous ties) or the chamber (transitive triads) to form independently. The interaction of these two components of echo chambers is a key component of information diffusion in policy networks. Understanding these processes will help us, in turn, make sense of the increasingly politically polarized world.
