# Correction: Shifting echo chambers in US climate policy networks

**DOI:** 10.1371/journal.pone.0274175

**Published:** 2022-09-01

**Authors:** The PLOS ONE Editors

The initials of the third and sixth authors are indexed incorrectly in PubMed. The correct initials are, respectively: Galli Robertson A; McCartney Waggle J.

The correct citation is: Jasny L, Dewey AM, Galli Robertson A, Yagatich W, Dubin AH, McCartney Waggle J, et al. (2018) Shifting echo chambers in US climate policy networks. PLoS ONE 13(9): e0203463. https://doi.org/10.1371/journal.pone.0203463

Additionally, the Data Availability statement for this paper is incorrect. The correct statement is: Data are available from the Climate Constituencies study at http://www.drfisher.umd.edu/CCP.html.

The publisher apologizes for the errors.
